# Successful Treatment of Streptococcal Toxic Shock Syndrome with Both Diffuse Peritonitis and Necrotizing Fasciitis

**DOI:** 10.1155/2018/8260968

**Published:** 2018-10-28

**Authors:** Shuichi Sato, Masahiro Ito, Tsuyoshi Sakai, Anri Kaneta, Fumie Sato

**Affiliations:** Department of Surgery, Kensei Hospital, Hirosaki-shi, Aomori, Japan

## Abstract

Streptococcal toxic shock syndrome (STSS) is a life-threatening disease caused by infection of *beta-hemolytic streptococci*. Here, we report an uncommon case of STSS with both diffuse peritonitis and necrotizing fasciitis and summarize previous cases. The patient was diagnosed with STSS due to an infection of the soft tissue of the lower extremity after surgery for diffuse peritonitis. The general condition had rapidly deteriorated with multiple organ dysfunction. Immediate intensive care, including mechanical ventilation, hemodiafiltration, and repeated debridement, is indispensable for a favorable outcome.

## 1. Introduction

Management of streptococcal toxic shock syndrome (STSS), a rare and life-threatening disease, requires immediate surgical intervention in addition to administration of appropriate antibiotics and intravenous immunoglobulin (IVIG) in an intensive care setting to achieve a favorable outcome. Here, we report our experience with a rare and extremely severe case of STSS combined with both diffuse peritonitis and necrotizing fasciitis (NF).

## 2. Case Presentation

**Figure 1:**
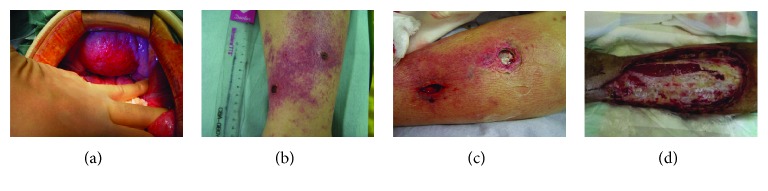
Case 1. (a, b) Admission day. Emergent laparotomy findings. The bladder wall and peritoneum showed remarkable redness and edematous changes (a). Left pretibial skin showed erythema and a few small scabs (b). (c) Four days after admission. The deeper section where scabs of the pretibial area were removed showed severe necrotic findings. (d) Two weeks after admission. After several rounds of debridement for NF at the pretibial area.

A 65-year-old woman with untreated diabetes visited our emergency department for a one-day history of lower abdominal pain. Abdominal findings showed diffuse peritonitis. Computed tomography showed edema of the small intestine and bladder wall. Perforation of the appendix, alimental tract, or urinary tract was suspected; thus, emergency surgery was performed. Although there were purulent ascites, remarkable redness, and edema of both the small intestine and bladder wall throughout the lower abdominal cavity, there was no perforation of the gastrointestinal or urinary tract (Figure 1(a)). Intraoperative Gram staining of purulent ascites in the abdominal cavity showed the presence of Gram-positive cocci. Based on these findings, idiopathic peritonitis was initially suspected; thus, only irrigation and drainage were performed to complete the surgery. Although a part of the patient's left lower leg appeared slightly red in the operating room, we mistakenly assumed that the presence of mild phlegmonous changes was not associated with the abdominal cavity findings. Postoperative monitoring of vital signs indicated that the patient was going into shock with respiratory failure and acidemia progression due to anuria despite administration of an inotropic agent and high-volume infusion of colloidal fluid. The maximum sequential organ failure assessment (SOFA) score was 10 points; thus, mechanical ventilation and carbapenem administration were initiated. Continuous hemodiafiltration (CHDF) was also necessary for renal support along with cytokine regulation due to prolonged anuria and acidemia. Infection and necrosis of the leg were gradually becoming more evident and severe (Figures 1(b) and 1(c)). Therefore, a diagnosis of NF was made.

*Group A streptococcus* (GAS) was detected in the ascites, blood culture, and purulent effusion from the left leg. Based on the above findings, the patient was diagnosed with STSS. The antibiotic regimen was changed to high-dose penicillin G and clindamycin to target the bacterial infection. Immunoglobulin was also administered. In addition, repeated debridement (Figure 1(d)) and skin grafting were performed. These aggressive therapeutic interventions gradually improved her general condition and the NF of her leg. She was discharged 6 months after hospitalization and is alive with well-controlled diabetes and with chronic hemodialysis at 7 years after this clinical course.

## 3. Discussion

STSS is a rapidly progressive and fatal disease caused by beta-hemolytic streptococcus infection with GAS as the most common pathogen [1]. STSS is similar to toxic shock syndrome, which is a well-known life-threatening disorder characterized by multiple organ dysfunction due to infection by *Staphylococcus aureus*. The representative risk factors for STSS are diabetes mellitus, alcohol dependence syndrome, malignant tumors, infection by human immunodeficiency virus, heart disease, and addiction to narcotic drugs [2]. The incidence of STSS continues to increase annually with a frequency of 0–3 cases per 1,000,000 inhabitants per year during the past 10 years in Aomori Prefecture, Japan. STSS is definitively diagnosed by the detection of hemolytic streptococcus in aseptic sites along with shock, organ failure, disseminated intravascular coagulation, soft tissue inflammation or necrosis, whole-body erythema, and central neurologic symptoms, such as the loss of consciousness and/or seizures [3, 4]. The clinical course is occasionally extraordinarily rapid and can be fatal within 24 h. When encountering soft tissue infection with the above described clinical symptoms, immediate empiric intervention is necessary despite a definitive diagnosis.

Our case presented with the unusual features of both diffuse peritonitis and NF. The maximum SOFA score was 10 points at two hospital days. It represents an extremely life-threatening condition due to the high bacterial load and a subsequent extreme immune reaction. Advanced intensive supportive care and aggressive surgical intervention are indispensable for such cases. However, it is necessary to distinguish STSS with diffuse peritonitis from diffuse peritonitis caused by transvaginal infections in premenopausal females.

In the present case, there was no purulent subglossal inflammation or infection from the vaginal insertion of sanitary products. Further, because the infectious findings of the lower extremity gradually became more severe each day, the final diagnosis was secondary peritonitis accompanied by a soft tissue infection.

Malota et al. and Iitaka et al. summarized the clinical presentation of patients with GAS peritonitis [5, 6]. In most such cases, laparotomy or laparoscopic laparotomy is performed on suspicion of a perforation of the gastrointestinal tract or appendix or on suspicion of general peritonitis with unknown origin. Because these cases did not show significant findings, except for purulent ascites, careful intra-abdominal examination, washing, and drainage were performed. Some cases underwent appendectomy or resection of a higher inflammatory site (e.g., bowel or omentum).

Of such cases of GAS peritonitis, STSS with both diffuse peritonitis and NF is infrequent. According to a review report by Malota et al., the frequency of this clinical presentation is 6% (2/35) [5]. Two such cases were described in only one report [7]. Since no other previous English reports have described STSS with both diffuse peritonitis and NF, the present case is considered to be both rare and severe. A summary of these cases, including our case, is shown in Table 1. The use of IVIG and mechanical support and the long-term survival are unknown in the previous report. However, our patient has survived for more than 7 years after surgery. Our case suggests that appropriate medical treatment can achieve a favorable outcome in survival even after such a life-threatening disease.

The critical points of a therapeutic strategy for STSS are as follows:

1. Intensive care and supportive management
2. Appropriate administration of antibiotics and IVIG
3. Aggressive surgical intervention for infectious sites

The pathophysiology of STSS is similar to that of septic shock. Therefore, systemic management is based on the treatment regimen for septic shock. Specifically, immediate and intensive treatment with colloidal fluid resuscitation, inotropic agents, adequate alimental support, and mechanical ventilation is indispensable for a favorable outcome, if needed [8]. Several recent reports of CHDF, which was mandatory for organ support in our case, have shown a benefit for the management of hypercytokinemia and renal dysfunction in cases of sepsis [9]. We propose that this therapeutic strategy may be necessary for STSS accompanied by multiple organ dysfunction.

As a first-line antimicrobial therapy for NF with streptococcal infection, high-dose penicillin G and clindamycin are recommended [10]. If STSS is suspected based on the clinical findings and culture results from the soft tissue and aseptic sites, the antibiotic regimen should be immediately changed to both high-dose penicillin G and clindamycin. In cases with NF, beta-lactam agents are less useful due to the higher organism load (inoculum effect). However, clindamycin is unaffected by the bacterial phase and inoculum size. This agent has other characteristics that suppress the synthesis of bacterial exotoxins, accelerate phagocytosis by neutrophils or monocytes by preventing M protein production, and have a higher transmigration rate to the infectious site, including abscesses. Because of these effects, concurrent administration of both penicillin and clindamycin is more effective than penicillin alone [11, 12].

IVIG administration is a valuable adjunctive therapy for STSS and probably offers a survival benefit. There have been several reports of highly successful cases of IVIG administration for the treatment of STSS. IVIG contains broad-spectrum antibodies against streptococcal superantigens and M proteins. Furthermore, anti-inflammatory effects have also been recognized, which decrease the production of proinflammatory cytokines (e.g., tumor necrosis factor alpha, interluekin-1 alpha, and interluekin-6), downregulate the expression levels of chemokines and chemokine receptors, and neutralize superantigens [13, 14].

Appropriate surgical intervention, including aggressive debridement and drainage of necrotizing soft tissue infections or other infection sites, is an essential therapeutic strategy to achieve a successful outcome. Several reports have shown a 7–9-fold increased risk of death with inadequate or delayed initial debridement or surgical intervention. To suppress disease progression, surgical intervention must be performed as early as possible and should be repeated subsequently depending on the clinical course of the soft tissue or other infection [15–18]. In NF, soft tissue infections generally extend more deeply than can be judged from skin surface findings, which may lead to the underestimation of the actual infectious range. Therefore, additional attention is necessary in case of surgical intervention. In our case, we initially noticed a small erythema on the patient's left lower leg. The necrotizing site had spread further and deeper to the fascia of the left tibialis anterior muscle. Frequent debridement was needed to remove the necrotic tissues. In particular, further careful attention is necessary for cases with diabetes mellitus, which is a representative risk factor for STSS. Many patients with untreated or poorly controlled diabetes have diabetic neuropathy and are, therefore, less conscious of pain. Although pain is an essential sign of inflammation, patients with diabetic neuropathy have insufficient self-awareness of pain, which can result in delayed surgical intervention of the infectious sites. Our case also felt less pain. Hence, daily physical assessment by the medical staff is practical for early detection and intervention against inflammatory findings for the effective prevention of disease progression.

STSS is an extremely aggressive and life-threatening disease. When diffuse peritonitis with unknown origin is identified during the initial surgery, STSS must be considered. Further, both evaluation of soft tissue infection and preparation for deterioration of physical status are significant in such cases. If a diagnosis of STSS is likely, immediate and aggressive therapeutic interventions, including appropriate antibiotic administration, IVIG, and intensive care, are indispensable to achieve a successful treatment outcome. In addition, CHDF is essential as mechanical support for renal dysfunction and cytokine regulation. Further, repeated surgical intervention, depending on the status of the infection, is also necessary.

## Figures and Tables

**Table 1:** Reports on STSS with peritonitis and NF.

Author	Year	Age/sex	Details of laparotomy	NF	IVIG	Mechanical support	Prognosis after discharge
Monneuse et al. [7]	2010	N/A	At least washing and drainage	Axillary, chest, leg	N/A	N/A	Alive, at least 3 months
Monneuse et al. [7]	2010	N/A	At least washing and drainage	Nose, finger	N/A	N/A	Alive, at least 3 months
Present case	2018	65F	Washing and drainage	Leg	+	CHDF, ventilation	Alive, 7 years

Abbreviations: CHDF: continuous hemodiafiltration; IVIG: intravenous immunoglobulin; N/A: not applicable; NF: necrotizing fasciitis.
